# Childhood emotional abuse and adolescent anxiety: a moderated mediation model of bullying victimization, perceived teacher legitimacy, and negative legal emotion

**DOI:** 10.3389/fpsyg.2026.1803574

**Published:** 2026-06-05

**Authors:** Shuowang Wang, Weiwei Sun, Zhijing Hong, Haiqiong Huang, Shuhui Xu

**Affiliations:** 1 School of Education, Wenzhou University, Wenzhou, China; 2 Wenzhou Research Center for Family Tradition and Family Education, Wenzhou, China; 3 Taishun Yucai Primary School, Wenzhou, China; 4 Wenzhou Ouhai Niushan Experimental School, Wenzhou, China

**Keywords:** anxiety, bullying victimization, childhood emotional abuse, negative legal emotion, perceived teacher legitimacy

## Abstract

**Background:**

Childhood emotional abuse (CEA) is a robust risk factor for adolescent anxiety, yet the psychosocial mechanisms linking early emotional maltreatment to later internalizing problems remain insufficiently understood. This study examined whether bullying victimization mediates the association between CEA and anxiety, and whether perceived teacher legitimacy and negative legal emotion jointly moderate this pathway.

**Methods:**

A sample of 952 middle school students in mainland China completed measures of CEA, bullying victimization, anxiety, perceived teacher legitimacy, and negative legal emotion. Spearman correlations and moderated mediation analyses were conducted using PROCESS Model 60 with 5,000 bootstrap resamples, controlling for gender, grade, and parental education.

**Results:**

CEA was positively associated with bullying victimization and anxiety. Bullying victimization partially mediated the relationship between CEA and anxiety. Negative legal emotion significantly strengthened the path from CEA to bullying victimization, indicating amplified risk among adolescents who held distrustful or cynical views toward legal authority. Perceived teacher legitimacy moderated the association between bullying victimization and anxiety, such that higher levels of perceived legitimacy intensified the psychological impact of victimization, particularly among students with elevated negative legal emotion. Conditional indirect effects varied across moderator levels, highlighting a dual-stage, context-dependent risk process.

**Conclusions:**

The findings support a dual-moderated mediation model in which individual legal-emotional orientations and school-based authority perceptions jointly shape the developmental cascade from childhood emotional abuse to adolescent anxiety. Interventions should integrate trauma-informed bullying prevention, efforts to enhance procedural justice and perceived teacher legitimacy, and targeted programs to reduce negative legal emotion and rebuild institutional trust.

## 1 Introduction

Childhood emotional abuse (CEA) refers to sustained psychological harm inflicted through degrading, humiliating, or intimidating behaviors by caregivers or other adults (Bernstein and Fink, 1998). As a core form of child maltreatment embedded in maladaptive parent–child interaction patterns, CEA has been widely recognized as a chronic and pervasive developmental risk (Hart and Brassard, 1987; Glaser, 2002). Global epidemiological evidence underscores its high prevalence and public health significance (World Health Organization, 2014). Compared with other forms of maltreatment, CEA shows a particularly robust and enduring association with internalizing symptoms, especially anxiety, functioning as an independent and stable risk factor across development (Al-Fayez et al., 2012; Bendall et al., 2023; Hovens et al., 2012; Simon et al., 2009). Within the Chinese context, emotional abuse frequently co-occurs with physical abuse (Chen et al., 2023; Fu et al., 2018), while adolescence represents a sensitive developmental window for the emergence and escalation of anxiety disorders (Young et al., 2019). Although the link between childhood emotional abuse and anxiety is well established, existing research has rarely integrated school-level authority factors, such as perceived teacher legitimacy, and broader sociocultural influences, such as negative legal emotion, into a unified explanatory framework.

At both neurobiological and psychosocial levels, CEA may exert persistent effects that heighten vulnerability to anxiety. Neurobiologically, early emotional maltreatment has been associated with altered stress-related neural functioning, including heightened amygdala reactivity (Field, 2025). Psychosocially, shattered assumptions theory posits that early trauma undermines fundamental beliefs about self-worth, interpersonal safety, and world predictability (Janoff-Bulman, 1992). Such disruptions may increase adolescents' social insecurity and interpersonal sensitivity, thereby elevating their risk of becoming targets of bullying victimization. Empirical evidence consistently supports this pathway, showing that childhood emotional abuse predicts greater susceptibility to peer victimization during adolescence (Chen et al., 2023; Xiao et al., 2021; Zhang et al., 2022). Bullying victimization, defined as repeated exposure to intentional harm within power-imbalanced peer relationships (Olweus, 1993), has been robustly linked to internalizing outcomes, including anxiety and depression (Balluerka et al., 2023; Halliday et al., 2021; Sigurdson et al., 2015). Together, these findings suggest that bullying victimization may function as a key mediating mechanism through which early emotional abuse contributes to later anxiety.

Beyond peer processes, adolescents' psychological adjustment is embedded within school and institutional contexts. Perceived teacher legitimacy refers to students' judgments that teachers are entitled to exercise authority because they are experienced as fair, respectful, and trustworthy in everyday school interactions (Gouveia-Pereira et al., 2017; Tyler and Trinkner, 2017). Drawing on procedural justice theory, adolescents are more likely to regard authority as legitimate when authority figures make decisions fairly, treat them with dignity, and communicate trustworthy motives (Tyler, 2006; Fagan and Tyler, 2005). In school settings, higher perceived teacher legitimacy may strengthen institutional trust and increase adolescents' willingness to seek help from teachers or other school adults when bullying occurs (Gregory and Ripski, 2008; Eliot et al., 2010; Gregory et al., 2010). Relatedly, active teacher responses to bullying can shape students' perceptions of teachers' anti-bullying attitudes, reporting intentions, and expected classroom dynamics (Demol et al., 2020). Accordingly, perceived teacher legitimacy may function as a school-based protective factor in adolescents' responses to peer victimization. At the societal level, adolescents' legal attitudes also shape their responses to victimization. Negative legal emotion, conceptually related to legal cynicism, reflects distrust, alienation, and negative evaluations of legal institutions and authority (Fagan and Tyler, 2005; Trinkner and Cohn, 2014; Xu and Yan, 2022). Such orientations may weaken confidence in formal authority and legal recourse, thereby heightening vulnerability following victimization (Fagan and Tyler, 2005; Kirk and Papachristos, 2011).

**Figure 1:**
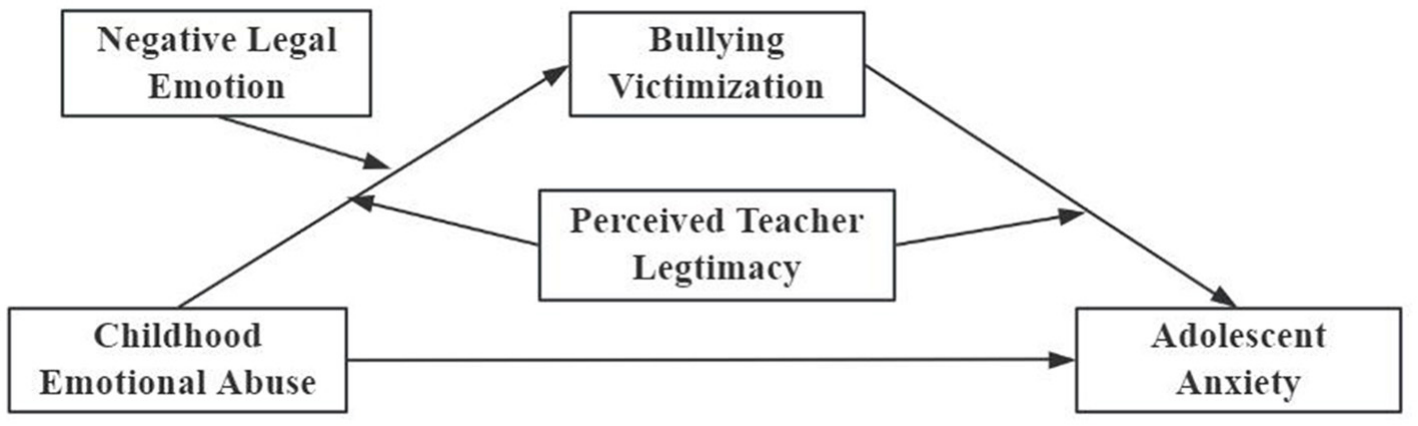
Hypothesized model.

Building on prior theory and evidence, this study proposes a moderated mediation model linking family adversity, peer processes, school authority, and legal attitudes. We hypothesize that bullying victimization mediates the association between childhood emotional abuse and adolescent anxiety (Hypothesis 1). Negative legal emotion is expected to moderate the first-stage path, such that higher legal cynicism strengthens the association between childhood emotional abuse and bullying victimization (Hypothesis 2). Perceived teacher legitimacy is expected to serve as a cross-stage moderator, shaping both the path from childhood emotional abuse to bullying victimization and the path from bullying victimization to adolescent anxiety (Hypothesis 3). By integrating interpersonal trauma, peer victimization, procedural justice, and institutional trust, this study aims to clarify when childhood emotional abuse increases risk for adolescent anxiety and to inform school-based interventions. The hypothesized model is shown in Figure 1.

## 2 Method

### 2.1 Participants and procedure

Data were collected in January 2026 from students at two secondary schools in mainland China using a convenience sampling strategy. A total of 1,000 questionnaires were distributed in classrooms and administered in a group-based paper-and-pencil format by trained researchers following a standardized protocol. The study was approved by the School of Education Research Ethics Committee of Wenzhou University (Approval No. WZUED20250401). Written informed consent was obtained from parents or legal guardians, and all students provided assent prior to participation. After data screening, cases showing patterned responding or missing key items were excluded. The final analytic sample consisted of 952 students, yielding a valid response rate of 95.2%.

The sample comprised 523 boys (54.9%) and 429 girls (45.1%). By grade, 37.1% were first-year students, 34.3% were second-year students, and 28.6% were third-year students. In mainland China, the school entry age for junior secondary students is relatively standardized; accordingly, students in first to third year typically fall within a narrow age range of approximately 13 to 15 years. Fathers' educational attainment was most commonly university (32.6%) or high school/technical secondary school (32.2%), followed by junior high school (26.5%), primary school or below (5.6%), and postgraduate education (3.2%); 0.3% reported no formal education. Mothers' educational attainment was also concentrated at the university (36.6%) and high school (30.5%) levels, followed by junior high school (23.9%), primary school or below (5.6%), and postgraduate education (3.0%), with only 0.4% reporting no formal education.

### 2.2 Measures

#### 2.2.1 Childhood trauma questionnaire-short form (CTQ-SF)

Childhood emotional abuse was assessed using the childhood trauma questionnaire-short form (CTQ-SF; Bernstein et al., 2003). The CTQ-SF consists of 28 items, including 25 clinical items and three validity items, and measures five domains: emotional abuse, physical abuse, sexual abuse, emotional neglect, and physical neglect. Items are rated on a five-point Likert scale (1 = never, 5 = always). In the present study, the scores of the five emotional abuse items were summed to index childhood emotional abuse, with higher scores indicating more severe experiences of emotional abuse during childhood. An example item is “People in my family said hurtful or insulting things to me.” The Chinese version used in this study was based on the revision by Zhao et al. (2005). We focused specifically on the emotional abuse subscale because the present study was concerned with the role of emotionally adverse childhood experiences, rather than childhood maltreatment as a global construct. This subscale was therefore considered to provide the closest conceptual match to the study hypotheses. The emotional abuse subscale showed acceptable internal consistency in the current sample (Cronbach's α = 0.708).

#### 2.2.2 Anxiety (PHQ-4 anxiety subscale)

Anxiety symptoms were measured using the two-item anxiety subscale of the patient health questionnaire-4 (PHQ-4; Löwe et al., 2010). The two items are “feeling nervous, anxious, or on edge” and “not being able to stop or control worrying.” Items are rated on a four point frequency scale (0 = not at all; 3 = nearly every day) and higher summed scores indicate more frequent anxiety symptoms. In the current study the anxiety subscale showed good internal consistency (Cronbach's α = 0.792).

#### 2.2.3 Perceived teacher legitimacy scale

Perceived teacher legitimacy was assessed using a 10-item scale adapted from the parental legitimacy measure developed by Trinkner et al. (2012) for use in the school context. The scale was designed to assess students' perceptions of the legitimacy of teacher authority, including their recognition of and voluntary compliance with teachers' authority. A sample item is, “Even if I do not like the way a teacher treats me, I should still follow the teacher's requests.” Responses were made on a 5-point Likert scale (1 = strongly disagree, 5 = strongly agree), with higher scores indicating higher perceived teacher legitimacy. It should be noted that this teacher-specific version has not been extensively validated in the Chinese context. In the present sample, the scale demonstrated modest internal consistency (Cronbach's α = 0.604), suggesting that its psychometric properties are limited and that results involving this measure should be interpreted cautiously.

#### 2.2.4 Bullying victimization scale

Bullying victimization was measured using the Chinese revision of the Olweus Bullying Questionnaire (Zhang et al., 1999). The seven-item scale assesses physical, verbal, relational, and cyber victimization over the past year. Items are rated on a five-point Likert scale, with higher scores indicating more frequent victimization. Example item: “Some classmates excluded me from activities, left me out of their peer group, or ignored me on purpose.” In the present study, the internal consistency of the bullying victimization scale was acceptable (Cronbach's α = 0.673).

#### 2.2.5 Negative legal emotion scale

Negative legal emotion was assessed using the 7-item Negative Legal Emotion Questionnaire for Secondary School Students developed and validated by Xu and Yan (2022). The scale assesses students' feelings of disappointment toward law and legal institutions. Items are rated on a five-point Likert scale (1 = strongly disagree, 5 = strongly agree), with higher scores indicating stronger negative legal emotion. An example item is “The law does not have much credibility in society.” Conceptually, negative legal emotion reflects an affective response to law and legal authority at a broader institutional level, whereas perceived teacher legitimacy refers to students' evaluations of the fairness, appropriateness, and legitimacy of teachers within the school context. Thus, although the two constructs are theoretically related, they represent distinct domains. In the current sample, the scale demonstrated excellent internal consistency (Cronbach's α = 0.892).

### 2.3 Data analysis

Data were analyzed using SPSS 25.0, IBM Corp., Armonk, NY, United States. Prior to hypothesis testing, we conducted data-quality checks, including assessments of missing data, outlier screening using boxplots and standardized z scores, and normality tests using the Kolmogorov–Smirnov and Shapiro-Wilk procedures. All core variables significantly deviated from normality (ps < 0.001). Therefore, Spearman rank-order correlations were used to examine bivariate associations. Given the large sample size and the use of bootstrapped confidence intervals in the main analyses, the original continuous scores were retained rather than transformed.

To enhance methodological transparency, hierarchical regression analyses were first conducted to examine the component paths of the hypothesized model in a stepwise manner. Predictors were entered in blocks to show the incremental contributions of the independent variable, mediator, moderator, and interaction terms to the relevant dependent variables. These analyses were intended to provide a transparent presentation of the regression steps underlying the model, rather than to serve as the sole basis for testing moderated mediation.

The hypothesized moderated mediation model was then formally tested using Hayes' PROCESS macro (Model 60; Hayes, 2018). This model was selected because the conceptual framework involved both mediation and moderation, and Model 60 enables these processes to be estimated simultaneously within a single analytic framework. This approach allows a more integrated test of the proposed mechanism by estimating direct, indirect, and conditional indirect effects concurrently. Gender, grade, and parental education were included as covariates to adjust for their potential confounding influences on the associations among the focal variables. Gender was included as a covariate to control for its potential confounding influence, whereas the primary analyses focused on testing the overall model in the full sample rather than gender-specific pathways. Grade, rather than age, was included because junior secondary students in mainland China typically enter school at a relatively standardized age, resulting in a narrow age range that is closely aligned with grade level. Under these conditions, grade serves as a reasonable proxy for age while also more directly reflecting the school-based developmental context. All continuous predictors were mean-centered prior to the construction of interaction terms. The significance of interaction terms and conditional indirect effects was evaluated using bias-corrected 95% bootstrap confidence intervals based on 5,000 resamples. Significant interactions were further probed using simple-slopes analyses. Variance inflation factors for all predictors were below three, indicating no evidence of serious multicollinearity.

## 3 Results

### 3.1 Descriptive statistics and bivariate associations

Descriptive statistics and Spearman rank-order correlations for all study variables are presented in Table 1. Participants were aged 13 to 15 years. All primary study variables were significantly intercorrelated (all ps < 0.01). Specifically, childhood emotional abuse was positively correlated with negative legal emotion (ρ = 0.340), bullying victimization (ρ = 0.295), and anxiety (ρ = 0.334), and negatively correlated with perceived teacher legitimacy (ρ = −0.185). Negative legal emotion was positively associated with bullying victimization (ρ = 0.250) and anxiety (ρ = 0.281), and negatively associated with perceived teacher legitimacy (ρ = −0.320). Bullying victimization was positively correlated with anxiety (ρ = 0.273). Perceived teacher legitimacy was negatively correlated with both bullying victimization and anxiety (ρ = −0.102 and ρ = −0.171, respectively).

**Table 1:** Descriptive statistics and correlations for key variables (*N* = 952).

Variable	*M* ±SD	1	2	3	4	5
1. CEA	6.88 ± 2.68	1				
2. NLE	12.58 ± 5.28	0.340^***^	1			
3. BV	8.49 ± 2.45	0.295^***^	0.250^***^	1		
4. Anxiety	1.80 ± 1.43	0.334^***^	0.281^***^	0.273^***^	1	
5. PTL	3.46 ± 0.45	−0.185^***^	−0.320^***^	−0.102^***^	−0.171^***^	1

^***^p < 0.001. ^***^ indicates statistical significance at the *p* < 0.001 level.

CEA, childhood emotional abuse; NLE, negative legal emotion; BV, bullying victimization; PTL, perceived teacher legitimacy.

**Figure 2:**
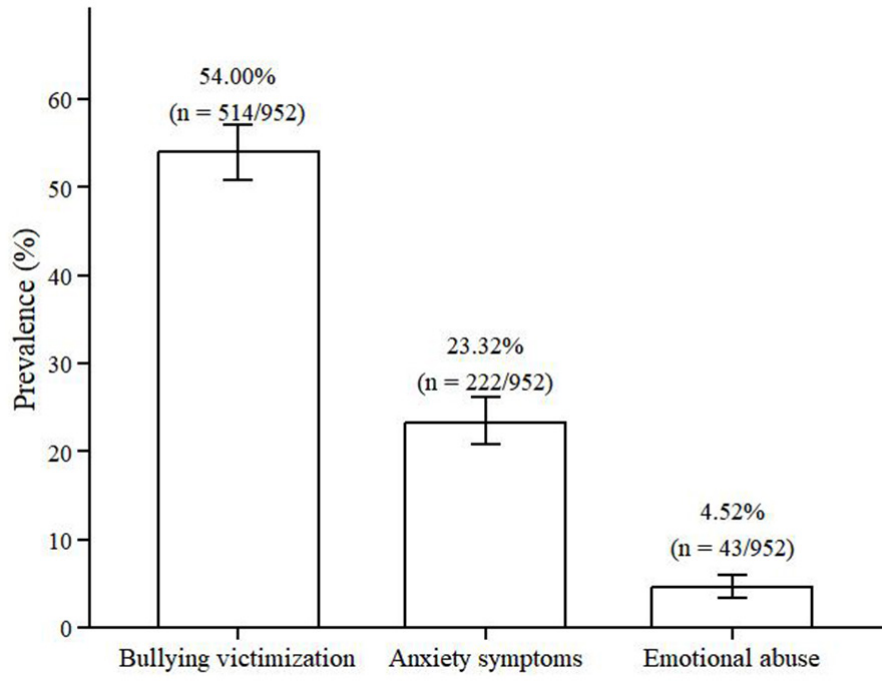
Prevalence of emotional abuse, anxiety symptoms, and bullying victimization among secondary school students.

With respect to prevalence, according to the cut-off criteria proposed by Bernstein and Fink (1998), the prevalence of emotional abuse was 4.52% (43/952). Based on the criterion suggested by Löwe et al. (2010), 23.32% of participants (222/952) screened positive for anxiety symptoms. According to the criterion reported by Zhang et al. (1999), 54.0% of participants (514/952) reported experiences of bullying victimization. These findings suggest that emotional abuse, anxiety symptoms, and bullying victimization were notable in this sample of secondary school students and therefore warrant attention (see Figure 2).

### 3.2 Tests of mediated and moderated effects

The hypothesized moderated mediation model was formally examined using Hayes' PROCESS macro for SPSS (Model 60). Childhood emotional abuse was entered as the predictor (X), bullying victimization as the mediator (M), anxiety as the outcome (Y), negative legal emotion as the moderator of the X → M path (Z), and perceived teacher legitimacy as the moderator of the M → Y path (W). Gender, grade, and parental education were included as covariates. Hierarchical regression analyses were additionally conducted to present the constituent paths of the model in a stepwise manner; however, inference regarding moderated mediation was based on the PROCESS estimates and bootstrap confidence intervals.

Results indicated that childhood emotional abuse significantly predicted anxiety directly (β = 0.270, *p* < 0.001). In addition, bullying victimization partially mediated this association: childhood emotional abuse significantly predicted bullying victimization (β = 0.253, *p* < 0.001), which in turn significantly predicted anxiety (β = 0.211, *p* < 0.001).

The moderation results further suggested stage-specific conditional effects. Negative legal emotion significantly moderated the path from childhood emotional abuse to bullying victimization (β = 0.063, *p* = 0.012), whereas perceived teacher legitimacy significantly moderated the path from bullying victimization to anxiety (β = 0.079, *p* < 0.001). These findings indicate that the indirect association between childhood emotional abuse and anxiety operated under different boundary conditions at different stages of the model. Specifically, the first-stage pathway was contingent on negative legal emotion, whereas the second-stage pathway was contingent on perceived teacher legitimacy. Full model coefficients and bootstrap-based conditional indirect effects are presented in Table 2.

**Table 2:** Results of moderated mediation regression analyses (*N* = 951).

Variable	Bullying victimization			Anxiety		
	β	SE	*t*	β	SE	*t*
Constant	0.06	0.163	0.368	0.112	0.164	0.679
CEA	0.253^***^	0.036	7.101	0.270^***^	0.033	8.22
Bullying victimization	—	—	—	0.211^***^	0.035	5.954
PTL	−0.044	0.032	−1.376	−0.071^*^	0.031	−2.314
NLE	0.116^***^	0.032	3.593	—	—	—
CEA × PTL	−0.037	0.024	−1.551	—	—	—
CEA × NLE	0.063^*^	0.025	2.507	—	—	—
BV × PTL	—	—	—	0.079^***^	0.023	3.453
Gender	0.355^***^	0.059	6.027	−0.306^***^	0.061	−5.058
Grade	−0.056	0.037	−1.514	0.092^*^	0.037	2.485
Mother's education	−0.013	0.038	−0.343	0	0.039	−0.007
Father's education	−0.022	0.038	−0.581	−0.034	0.038	−0.875
*R^2^*	0.205			0.185		
*F*	26.890^***^			26.701^***^		

^*^p < 0.05, ^***^p < 0.001.

CEA, childhood emotional abuse; NLE, negative legal emotion; BV, bullying victimization; PTL, perceived teacher legitimacy.

### 3.3 Simple-slopes and conditional indirect effects

Simple-slopes analyses were conducted to probe the significant interaction effects. For the first stage of the indirect pathway (childhood emotional abuse → bullying victimization), the positive association between childhood emotional abuse and bullying victimization became stronger as negative legal emotion increased. Specifically, the effect was strongest at +1 SD of negative legal emotion (effect = 0.317, *p* < 0.001), moderate at the mean level (effect = 0.253, *p* < 0.001), and weakest at −1 SD, although it remained significant (effect = 0.190, *p* < 0.001). These findings indicate that the association between childhood emotional abuse and bullying victimization was stronger among adolescents with higher levels of negative legal emotion.

For the second stage of the indirect pathway (bullying victimization → anxiety), simple-slopes analyses showed that perceived teacher legitimacy significantly moderated the association between bullying victimization and anxiety. Specifically, the positive association was stronger at higher levels of perceived teacher legitimacy (+1 SD; effect = 0.290, *p* < 0.001) than at lower levels (−1 SD; effect = 0.132, *p* < 0.001). Thus, the strength of the association between bullying victimization and anxiety varied as a function of perceived teacher legitimacy (see Supplementary Tables S1, S2).

Bootstrap analyses based on 5,000 resamples were then used to examine the conditional indirect effects (see Table 3). The indirect effect of childhood emotional abuse on anxiety through bullying victimization was significant under most combinations of the moderators, although its magnitude varied across conditions. In general, the indirect effect was stronger at mean and high levels of negative legal emotion. Two combinations yielded nonsignificant conditional indirect effects: low perceived teacher legitimacy combined with low negative legal emotion, and high perceived teacher legitimacy combined with low negative legal emotion. Overall, these results suggest that the indirect association between childhood emotional abuse and anxiety via bullying victimization was contingent on the joint levels of negative legal emotion and perceived teacher legitimacy.

**Table 3:** Analysis of conditional indirect effects.

Perceived teacher legitimacy	Negative legal emotion	Indirect effect	Boot SE	Boot 95% CI
Low (−1 SD)	Low (−1 SD)	0.03	0.016	[−0.0003,0.063]
Low (−1 SD)	Mean (0)	0.038^*^	0.013	[0.013,0.065]
Low (−1 SD)	High (+1 SD)	0.047^*^	0.018	[0.013,0.082]
Mean (0)	Low (−1 SD)	0.040^*^	0.025	[0.002,0.099]
Mean (0)	Mean (0)	0.053^***^	0.014	[0.031,0.084]
Mean (0)	High (+1 SD)	0.067^***^	0.018	[0.032,0.104]
High (+1 SD)	Low (−1 SD)	0.044	0.04	[−0.005,0.141]
High (+1 SD)	Mean (0)	0.063^***^	0.023	[0.032,0.121]
High (+1 SD)	High (+1 SD)	0.081^***^	0.025	[0.034,0.135]

^*^p < 0.05, ^***^p < 0.001.

Indirect path: childhood emotional abuse (*X*) → bullying victimization (*M*) → anxiety (*Y*).

## 4 Discussion

### 4.1 Bullying victimization as a mediator: psychosocial transmission from family risk to peer harm

Childhood emotional abuse operates as an early developmental risk that is transmitted to later peer victimization and subsequent maladaptive adjustment through multiple psychosocial mechanisms. First, emotional abuse undermines self-concept, emotion-regulation capacities, and social skills, fostering social withdrawal or dysregulated behaviors that increase vulnerability to peer attack; this pattern is evident across studies of traditional and cyber bullying (Geng et al., 2022; Benedini et al., 2016; Yoon et al., 2018). Second, prior maltreatment may incline adolescents to affiliate with antisocial peers. Such peer contexts often fail to protect victims and may instead normalize aggression or downplay victimization, thereby increasing the risk of bullying. This effect is further amplified when student–teacher relationships are weak and adult support is limited (Wang et al., 2024). Third, bullying victimization itself constitutes a recurring interpersonal trauma that activates stress-response systems and consolidates negative cognitive schemas, which in turn sustain and amplify internalizing symptoms such as anxiety (Balluerka et al., 2023; Halliday et al., 2021). Taken together, bullying victimization functions not merely as an outcome of early family adversity but as a key mediating process that links childhood emotional abuse to longer-term psychosocial difficulties. This underscores the need for interventions addressing not only individual-level sequelae of maltreatment but also the peer ecology and school support structures that permit victimization to persist and to cascade into enduring mental-health problems.

### 4.2 The moderating role of negative legal emotion in the association between childhood emotional abuse and bullying victimization

The moderation analyses indicate that negative legal emotion strengthens the predictive association between childhood emotional abuse and subsequent bullying victimization. This finding is consistent with perspectives integrating legal socialization and emotion socialization (He et al., 2025; Xu et al., 2024). Negative legal emotion, reflected in distrust, alienation, and unfavorable evaluations of law and authority (Xu and Yan, 2022; He et al., 2025; Wang et al., 2025), appears to function as a cognitive and affective filter. Adolescents with elevated negative legal emotion are more likely to interpret interpersonal conflicts through hostile attributional biases or to adopt avoidant responses, thereby increasing their vulnerability to peer victimization. At a mechanistic level, heightened negative legal emotion may signal a generalized skepticism toward rules and fairness, which can diminish the motivation to seek formal protection or to engage in proactive coping when confronted with bullying. Prior research indicates that legal cynicism undermines collective efficacy and reduces access to formal sources of support (Fagan and Tyler, 2005; Kirk and Papachristos, 2011). In addition, converging evidence from research on victim–perpetrator trajectories indicates that moral disengagement increases the likelihood that victimization will escalate into aggressive behavior, whereas stronger legal cognition serves a protective function by mitigating this transition (Shuhui et al., 2025).

Taken together, adolescents' cognitive and emotional orientations toward law and institutional authority, as captured by negative legal emotion, play a substantive role in shaping post victimization behavioral and psychological adaptation. Rather than constituting a mere outcome of adversity, negative legal emotion operates as a risk amplifier that increases the likelihood that early family trauma will translate into peer victimization and, ultimately, anxiety.

### 4.3 Conditional role of perceived teacher legitimacy at the downstream stage: environmental sensitivity and cognitive congruence

The present findings indicate that perceived teacher legitimacy exerts a context-dependent moderating effect on the pathway from bullying victimization to anxiety. At a general level, higher perceived teacher legitimacy and procedural justice are typically associated with reduced aggressive behavior and stronger endorsement of school norms, thereby functioning as an important environmental protective factor (Xu and Chen, 2023). This pattern is consistent with prior research on school climate. For example, Gage et al. (2014), using a latent class growth model, found that a positive school climate, particularly teacher support, significantly reduces bullying victimization. However, this protective effect appears developmentally contingent: teacher support exerts a more direct influence among younger students, whereas among adolescents it operates more effectively in conjunction with peer support systems. This further highlights the situational and developmental specificity of the protective function of teacher authority.

Notably, among adolescents with high levels of negative legal emotion (legal cynicism), a highly authoritative school environment may paradoxically intensify anxiety following bullying victimization, reflecting a cognitive–environmental incongruence effect. This pattern can be interpreted through an integrative lens combining legal socialization theory and individual differences in environmental sensitivity. First, Kaiser and Reisig (2019) demonstrated that legal cynicism is a stable predictor of problem behaviors and that the effects of procedural justice depend on the internalization of legitimacy. When students have already developed entrenched distrust toward institutional authority, externally imposed high-authority structures are unlikely to be internalized as legitimate sources of protection. Instead, bullying incidents occurring in such environments may be interpreted as evidence of systemic failure, reinforcing preexisting negative beliefs and eliciting heightened psychological distress, consistent with a “disappointment under high expectations” mechanism.

Moreover, legitimacy is not grounded solely in formal procedural fairness but also in the extent to which authority conveys respect, recognition, and interpersonal dignity in everyday interactions (Oliveira and Jackson, 2021). For students high in negative legal emotion, perceived failure of authorities to intervene effectively in bullying or to demonstrate substantive fairness may constitute a form of legitimacy breakdown, further eroding institutional trust and solidifying negative self- and world-views following victimization.

In addition, individuals differ markedly in their sensitivity to environmental influences (Pluess, 2015). Adolescents with elevated negative legal emotion may have experienced repeated exposure to maltreatment, neglect, or injustice, shaping heightened psychophysiological reactivity to cues of threat and unfairness. As a result, they may be more attuned to inconsistencies in procedures, insufficient support, or delayed institutional responses within high-authority environments, leading to deeper emotional and cognitive engagement and an amplified anxiety response.

Taken together, these findings suggest that the effects of perceived teacher legitimacy are not uniform but are contingent upon students' preexisting legal–emotional orientations and their sensitivity to environmental signals.

### 4.4 Dual moderation and intervention implications

Overall, negative legal emotion and perceived teacher legitimacy appear to exert complementary influences at different stages of the risk chain. Negative legal emotion primarily shapes the translation of early family adversity into peer victimization, whereas perceived teacher legitimacy determines whether victimization escalates into anxiety and other internalizing outcomes. This dual-moderation pattern indicates that adolescents' affective orientations toward rules and authority not only shape their perception of social risk but also interact dynamically with institutional authority structures in schools to influence developmental trajectories of psychological adjustment.

Based on these findings, intervention strategies should target three key domains. First, adolescents with elevated negative legal emotion may benefit from interventions emphasizing legal education, narrative-based approaches, and trust-repair strategies to improve their cognitive and affective orientations toward authority and rules, thereby strengthening their psychological connection to institutional fairness and formal support systems (Tyler, 2006; Tyler and Trinkner, 2017). Second, efforts to enhance perceived teacher legitimacy and procedural justice should be accompanied by increased transparency, participatory opportunities, and interpersonal respect to prevent defensive reactions or psychological backlash in contexts characterized by high authority but low trust (Trinkner et al., 2012; Fine and van Rooij, 2021). Third, bullying prevention should integrate family, peer, and school systems by identifying childhood emotional abuse early and providing structured emotion-socialization training and peer-mediation support to interrupt the cascading transmission of risk across ecological levels (Arseneault, 2017; Espelage et al., 2013; Moore et al., 2017).

### 4.5 Limitations and future directions

Several limitations should be acknowledged. First, the cross-sectional design precludes causal inference regarding the ordering of childhood emotional abuse, bullying victimization, and anxiety. Longitudinal and experimental studies are needed to test the temporal and directional assumptions of the proposed model. Second, all variables were measured by self-report, which may have introduced common method bias; future studies should incorporate multi-informant, behavioral, or physiological indicators. Third, although gender was included as a covariate and grade was used as a proxy for age, the present study did not formally examine gender-specific pathways or the potential contribution of finer age variation. Future research should test whether the proposed model operates differently across gender and developmental stages. Fourth, the psychometric support for the perceived teacher legitimacy measure was limited in the present sample, including modest internal consistency and suboptimal CFA results, and the teacher-specific version has not been fully validated in the Chinese context. In addition, because the sample was drawn from a single Chinese site and only the emotional abuse domain of the CTQ-SF was examined, caution is needed in generalizing these findings across contexts and across other forms of childhood maltreatment.

## 5 Conclusion

This study identifies a conditional pathway linking childhood emotional abuse to adolescent anxiety through bullying victimization, showing that this process is shaped by both negative legal emotion and perceived teacher legitimacy. Negative legal emotion strengthens the conversion of early family adversity into peer victimization, whereas perceived teacher legitimacy influences whether victimization escalates into anxiety in ways that depend on adolescents' underlying legal attitudes. By integrating legal socialization with school climate perspectives, these findings extend developmental models of victimization and internalizing symptoms. The results underscore the importance of interventions that enhance institutional trust and promote procedurally fair and legitimate school authority. Future longitudinal and experimental research is needed to establish causal mechanisms and refine targeted, context sensitive prevention strategies.

## Data Availability

The raw data supporting the conclusions of this article will be made available by the authors, without undue reservation.

## References

[B1] Al-Fayez G. A. Ohaeri J. U. Gado O. M. (2012). Prevalence of physical, psychological, and sexual abuse among a nationwide sample of Arab high school students: association with family characteristics, anxiety, depression, self-esteem, and quality of life. Soc. Psychiatry Psychiatr. Epidemiol. 47, 53–66. doi: 10.1007/s00127-010-0311-2 21076913

[B2] Arseneault L. (2017). The long-term impact of bullying victimization on mental health. World Psychiatry 16, 27–28. doi: 10.1002/wps.20399 28127927 PMC5269482

[B3] Balluerka N. Aliri J. Goñi-Balentziaga O. Gorostiaga A. (2023). Association between bullying victimization, anxiety and depression in childhood and adolescence: the mediating effect of self-esteem. Rev. Psicodidact. (Engl. Ed.) 28, 26–34. doi: 10.1016/j.psicoe.2022.11.001

[B4] Bendall S. Eastwood O. Spelman T. McGorry P. Hickie I. Yung A. R. . (2023). Childhood trauma is prevalent and associated with co-occurring depression, anxiety, mania and psychosis in young people attending Australian youth mental health services. Aust. N. Z. J. Psychiatry 57, 1518–1526. doi: 10.1177/00048674231177223 37243364

[B5] Benedini K. M. Fagan A. A. Gibson C. L. (2016). The cycle of victimization: the relationship between childhood maltreatment and adolescent peer victimization. Child Abuse Negl. 59, 111–121. doi: 10.1016/j.chiabu.2016.08.003 27568065

[B6] Bernstein D. P. Fink L. (1998). Childhood Trauma Questionnaire Manual. San Antonio, TX: Harcourt Brace and Company.

[B7] Bernstein D. P. Stein J. A. Newcomb M. D. Walker E. Pogge D. Ahluvalia T. . (2003). Development and validation of a brief screening version of the childhood trauma questionnaire. Child Abuse Negl. 27, 169–190. doi: 10.1016/S0145-2134(02)00541-0 12615092

[B8] Chen X. Shao J. Pu X. Wang Z. (2023). Childhood maltreatment and adolescents' peer victimization: the effect of security, school connectedness and gender. Child. Youth Serv. Rev. 148:106843. doi: 10.1016/j.childyouth.2023.106843

[B9] Demol K. Verschueren K. Salmivalli C. Colpin H. (2020). Perceived teacher responses to bullying influence students' social cognitions. Front. Psychol. 11:592582. doi: 10.3389/fpsyg.2020.592582 33335501 PMC7735982

[B10] Eliot M. Cornell D. Gregory A. Fan X. (2010). Supportive school climate and student willingness to seek help for bullying and threats of violence. J. Sch. Psychol. 48, 533–553. doi: 10.1016/j.jsp.2010.07.001 21094397

[B11] Espelage D. L. Low S. Polanin J. R. Brown E. C. (2013). The impact of a middle school program to reduce aggression, victimization, and sexual violence. J. Adolesc. Health 53, 180–186. doi: 10.1016/j.jadohealth.2013.02.021 23643338

[B12] Fagan J. Tyler T. R. (2005). Legal socialization of children and adolescents. Soc. Justice Res. 18, 217–241. doi: 10.1007/s11211-005-6823-3

[B13] Field T. (2025). Emotional abuse research: a narrative review. J. Clin. Psychol. Neurol. 3, 1–13. doi: 10.61440/JCPN.2025.v3.53

[B14] Fine A. D. van Rooij B. (2021). Legal socialization: understanding the obligation to obey the law. J. Soc. Issues 77, 367–391. doi: 10.1111/josi.12440

[B15] Fu H. Feng T. Qin J. Wang T. Wu X. Cai Y. . (2018). Reported prevalence of childhood maltreatment among Chinese college students: a systematic review and meta-analysis. PLoS One 13:e0205808. doi: 10.1371/journal.pone.0205808 30321243 PMC6188789

[B16] Gage N. A. Prykanowski D. A. Larson A. (2014). School climate and bullying victimization: a latent class growth model analysis. Sch. Psychol. Q. 29, 256–271. doi: 10.1037/spq0000064 24933216

[B17] Geng J. Bao L. Wang H. Wang J. Wei X. Lei L. (2022). The relationship between childhood maltreatment and adolescents' cyberbullying victimization: the new phenomenon of a “cycle of victimization.” *Child Abuse Negl*. 134:105888. doi: 10.1016/j.chiabu.2022.105888 36152532

[B18] Glaser D. (2002). Emotional abuse and neglect (psychological maltreatment): a conceptual framework. Child Abuse Negl. 26, 697–714. doi: 10.1016/S0145-2134(02)00342-3 12201163

[B19] Gouveia-Pereira M. Vala J. Correia I. (2017). Teachers' legitimacy: effects of justice perception and social comparison processes. Br. J. Educ. Psychol. 87, 1–15. doi: 10.1111/bjep.12131 27743388

[B20] Gregory A. Cornell D. Fan X. Sheras P. Shih T.-H. Huang F. (2010). Authoritative school discipline: high school practices associated with lower bullying and victimization. J. Educ. Psychol. 102, 483–496. doi: 10.1037/a0018562

[B21] Gregory A. Ripski M. B. (2008). Adolescent trust in teachers: implications for behavior in the high school classroom. Sch. Psych. Rev. 37, 337–353. doi: 10.1080/02796015.2008.12087881

[B22] Halliday S. Gregory T. Taylor A. Digenis C. Turnbull D. (2021). The impact of bullying victimization in early adolescence on subsequent psychosocial and academic outcomes across the adolescent period: a systematic review. J. Sch. Violence 20, 351–373. doi: 10.1080/15388220.2021.1913598

[B23] Hart S. N. Brassard M. R. (1987). A major threat to children's mental health: psychological maltreatment. Am. Psychol. 42, 160–165. doi: 10.1037/0003-066X.42.2.160 3578994

[B24] Hayes A. F. (2018). Introduction to Mediation, Moderation, and Conditional Process Analysis: A Regression-Based Approach, 2nd Edn. New York, NY: Guilford Press.

[B25] He J. Su X. Xu S. (2025). Parent–child attachment and adolescent problematic behavior: the mediating effect of legal emotions. Front. Psychol. 16:1546895. doi: 10.3389/fpsyg.2025.1546895 40083762 PMC11903415

[B26] Hovens J. M. Giltay E. J. Wiersma J. E. Spinhoven P. P. Penninx B. H. Zitman F. G. (2012). Impact of childhood life events and trauma on the course of depressive and anxiety disorders. Acta Psychiatr. Scand. 126, 198–207. doi: 10.1111/j.1600-0447.2011.01828.x 22268708

[B27] Janoff-Bulman R. (1992). Shattered Assumptions: Towards a New Psychology Of Trauma. New York, NY: Free Press.

[B28] Kaiser K. Reisig M. D. (2019). Legal socialization and self-reported criminal offending: the role of procedural justice and legal orientations. J. Quant. Criminol. 35, 135–154. doi: 10.1007/s10940-017-9375-4

[B29] Kirk D. S. Papachristos A. V. (2011). Cultural mechanisms and the persistence of neighborhood violence. Am. J. Sociol. 116, 1190–1233. doi: 10.1086/655754 21648250

[B30] Löwe B. Wahl I. Rose M. Spitzer C. Glaesmer H. Wingenfeld K. . (2010). A 4-item measure of depression and anxiety: validation and standardization of the Patient Health Questionnaire-4 (PHQ-4) in the general population. J. Affect. Disord. 122, 86–95. doi: 10.1016/j.jad.2009.06.019 19616305

[B31] Moore S. E. Norman R. E. Suetani S. Thomas H. J. Sly P. D. Scott J. G. (2017). Consequences of bullying victimization in childhood and adolescence: a systematic review and meta-analysis. World J. Psychiatry 7, 60–76. doi: 10.5498/wjp.v7.i1.60 28401049 PMC5371173

[B32] Oliveira T. R. Jackson J. (2021). Legitimacy, trust and legal cynicism: a review of concepts. Tempo Soc. 33, 113–145. doi: 10.11606/0103-2070.ts.2021.191381

[B33] Olweus D. (1993). Bullying at School: What We Know and What We Can Do. Oxford: Blackwell.

[B34] Pluess M. (2015). Individual differences in environmental sensitivity. Child Dev. Perspect. 9, 138–143. doi: 10.1111/cdep.12120

[B35] Shuhui X. Yu Z. Chunjing S. Zhiqiang W. (2025). From victim to bully: unpacking moral disengagement and the buffering effect of legal cognition. BMC Public Health 25:4015. doi: 10.1186/s12889-025-25364-7 41254641 PMC12625178

[B36] Sigurdson J. F. Undheim A. M. Wallander J. L. Lydersen S. Sund A. M. (2015). The long-term effects of being bullied or a bully in adolescence on externalizing and internalizing mental health problems in adulthood. Child Adolesc. Psychiatry Ment. Health 9:42. doi: 10.1186/s13034-015-0075-2 26300969 PMC4546259

[B37] Simon N. M. Herlands N. N. Marks E. H. Mancini C. Letamendi A. Li Z. . (2009). Childhood maltreatment linked to greater symptom severity and poorer quality of life and function in social anxiety disorder. Depress. Anxiety 26, 1027–1032. doi: 10.1002/da.20604 19750554 PMC2991116

[B38] Trinkner R. Cohn E. S. (2014). Putting the “social” back in legal socialization: procedural justice, legitimacy, and cynicism in legal and nonlegal authorities. Law Hum. Behav. 38, 602–617. doi: 10.1037/lhb0000107 25243981

[B39] Trinkner R. Cohn E. S. Rebellon C. J. Van Gundy K. (2012). Don't trust anyone over 30: parental legitimacy as a mediator between parenting style and changes in delinquent behavior over time. J. Adolesc. 35, 119–132. doi: 10.1016/j.adolescence.2011.05.003 21669454

[B40] Tyler T. R. (2006). Why People Obey the Law, 2nd Edn. Princeton, NJ: Princeton University Press. doi: 10.1515/9781400828609

[B41] Tyler T. R. Trinkner R. (2017). Why Children Follow Rules: Legal Socialization and the Development of Legitimacy. Oxford: Oxford University Press. doi: 10.1093/acprof:oso/9780190644147.001.0001

[B42] Wang X. Tian F. Wang P. (2024). Childhood psychological maltreatment predicts adolescents' bullying victimization: deviant peer affiliation and teacher–student relationships as moderators. Child. Youth Serv. Rev. 163:107814. doi: 10.1016/j.childyouth.2024.107814

[B43] Wang Z. Q. Zhang M. Xu S. H. (2025). The moderating role of legal emotions in the relationship between sensation seeking and risk-taking behaviors among college students. Front. Psychol. 16:1605528. doi: 10.3389/fpsyg.2025.1605528 40777220 PMC12330210

[B44] World Health Organization (2014). Global Status Report on Violence Prevention 2014. World Health Organization. Available online at: https://www.who.int/publications/i/item/9789241564793 (Accessed May 12, 2026).

[B45] Xiao Y. Jiang L. Yang R. Ran H. Wang T. He X. . (2021). Childhood maltreatment with school bullying behaviors in Chinese adolescents: a cross-sectional study. J. Affect. Disord. 281, 941–948. doi: 10.1016/j.jad.2020.11.022 33220948

[B46] Xu H. Chen Z. (2023). Perceived teacher procedural justice and aggressive behaviors among Chinese primary students: the mediating roles of negative evaluation of school rules and malicious envy. Soc. Psychol. Educ. 26, 25–44. doi: 10.1007/s11218-022-09737-z

[B47] Xu S. Yan W. (2022). The Development Characteristics, Influencing Factors and Mechanism of Adolescents' Legal Consciousness. Hangzhou: Zhejiang University Press.

[B48] Xu S. Yu J. Fan L. Yang Q. Wang Z. Zhang Y. (2024). The influencing factors of college students' legal emotion and the mechanism of its effect on aggressive behavior. Front. Psychol. 15:1295915. doi: 10.3389/fpsyg.2024.1295915 38699570 PMC11063308

[B49] Yoon D. Yoon S. Park J. Yoon M. (2018). A pernicious cycle: finding the pathways from child maltreatment to adolescent peer victimization. Child Abuse Negl. 81, 139–148. doi: 10.1016/j.chiabu.2018.04.024 29734111

[B50] Young K. S. Sandman C. F. Craske M. G. (2019). Positive and negative emotion regulation in adolescence: links to anxiety and depression. Brain Sci. 9:76. doi: 10.3390/brainsci9040076 30934877 PMC6523365

[B51] Zhang H. Han T. Ma S. Qu G. Zhao T. Ding X. . (2022). Association of child maltreatment and bullying victimization among Chinese adolescents: the mediating role of family function, resilience, and anxiety. J. Affect. Disord. 299, 12–21. doi: 10.1016/j.jad.2021.11.053 34822918

[B52] Zhang W. Wu J. Jones K. (1999).Revision of the Chinese version of the Olweus Child Bullying Questionnaire. Psychol. Dev. Educ. 15, 7–11.

[B53] Zhao X. Zhang Y. Li L. Zhou Y. Li H. Yang S. (2005). Reliability and validity of the Chinese version of the Childhood Trauma Questionnaire. Chin. J. Clin. Rehabil. 9, 105–107.

