# Supplementary material for: Childhood emotional abuse and adolescent anxiety: a moderated mediation model of bullying victimization, perceived teacher legitimacy, and negative legal emotion
Source: Front Psychol. 2026 Jun 5;17:1803574. doi: 10.3389/fpsyg.2026.1803574 (PMC13278893; doi:10.3389/fpsyg.2026.1803574)
Supplement: Supplementary file 1 [file Table_1.docx]

### ****Table S1.** Moderating Effect at Stage 1 (X → M)**

| Negative Legal Emotion | Effect (X→M) | *SE* | *t* | 95% CI |
| --- | --- | --- | --- | --- |
| Low (-1 SD) | .190^***^ | 0.051 | 3.762 | [.091， .289] |
| Mean (0) | .253^***^ | 0.036 | 7.101 | [.183， .323] |
| High (+1 SD) | .317^***^ | 0.036 | 8.879 | [.247， .387] |

### ^***^p<.001.

### ****Table S2.** Moderating Effect at Stage 1 (M → Y)**

| Teacher Legitimacy | Effect (M→Y) | *SE* | *t* | *95% CI* |
| --- | --- | --- | --- | --- |
| Low (-1 SD) | .132^***^ | 0.034 | 3.919 | [.066, .197] |
| Mean (0) | .211^***^ | 0.035 | 5.954 | [.141, .280] |
| High (+1 SD) | .290^***^ | 0.049 | 5.878 | [.193, .387] |

### ^***^p < .001.
